# Impact of Weekly Part-Time Infectious Disease Specialist Interventions and Collaboration with an Antimicrobial Stewardship Team in a Medium-Sized Community Hospital in Japan

**DOI:** 10.31662/jmaj.2025-0170

**Published:** 2025-09-12

**Authors:** Yoshiro Hadano, Ryuichi Oku, Mika Yanagihara, Junko Nakabayashi, Kumiko Takahashi, Sanae Sota, Yasuhiko Yokoyama

**Affiliations:** 1 Division of Infection Control and Prevention, Shimane University Hospital, Izumo, Shimane, Japan; 2 Antimicrobial stewardship team, Matsue Seikyo General Hospital, Matsue, Shimane, Japan; 3 Department of Pharmacy, Matsue Seikyo General Hospital, Matsue, Shimane, Japan; 4 Department of Nursing, Matsue Seikyo General Hospital, Matsue, Shimane, Japan; 5 Department of Clinical Laboratory, Matsue Seikyo General Hospital, Matsue, Shimane, Japan; 6 Department of Surgery, Matsue Seikyo General Hospital, Matsue, Shimane, Japan

**Keywords:** antimicrobial stewardship, *Staphylococcus aureus* bacteremia, antimicrobial resistance, infectious disease specialist, community hospital

## Introduction

The rise of drug-resistant bacteria, driven by inappropriate antimicrobial use and the slowdown in new drug development, has made antimicrobial resistance (AMR) a global public health concern ^(1)^. This highlights the growing importance of antimicrobial stewardship programs (ASPs) in combating AMR ^(2)^. The IDSA recommends including certified infectious disease (ID) physicians and pharmacists as core ASP members ^(3)^, yet many hospitals face workforce shortages. In Japan, medium-sized hospitals often lack ID specialists, and ASPs are frequently implemented without their direct involvement ^(4)^. Despite having fewer ID specialists than other countries, Japan’s infection control teams and antimicrobial stewardship teams (ASTs) have addressed these challenges effectively ^(5), (6)^. Collaboration with part-time ID specialists may help bridge the gap. Although studies in Japan have shown the benefits of weekly part-time ID interventions in tertiary hospitals ^(7), (8)^, evidence is limited for small to medium-sized or community hospitals, where trained AST staff are fewer. This study evaluates changes in broad-spectrum antimicrobial use associated with weekly part-time involvement of an ID specialist in collaboration with the hospital’s AST, as well as adherence to management bundles for *Staphylococcus aureus* bacteremia (SAB).

## Methods

This single-center retrospective study was conducted at Matsue Seikyo General Hospital (MSGH), a 351-bed community hospital in Matsue, Shimane, Japan. This hospital includes the following departments: Internal Medicine, Cardiology, Gastroenterology, Respiratory Medicine, Neurology, Surgery, Orthopedic Surgery, Obstetrics and Gynecology, Urology, Neurosurgery, Otolaryngology, Rehabilitation, Ophthalmology, Breast Surgery, Nephrology, Dermatology, and Radiology. This hospital provides treatment for some gastrointestinal tumors but generally does not manage cancers in other organ systems. In April 2022, a board-certified ID specialist began working 4 hours per week, attending AST conferences, and providing ID consultations for outpatients and inpatients. At MSGH, the AST was established in 2018 and has been active since then. A multidisciplinary ASP was conducted by a surgeon (0.1 full-time equivalent [FTE]), two clinical pharmacists (0.2 FTE), a microbiology technician (0.1 FTE), an infection control nurse (0.1 FTE), and, during the intervention period, a part-time ID physician (0.1 FTE). The ID specialist participated in a weekly 1.5-hour AST case conference, where AST members reviewed cases and supported appropriate antimicrobial use. The focus was on patients receiving broad-spectrum agents (carbapenems, piperacillin/tazobactam), those with positive blood cultures, and difficult-to-treat, defined primarily as those referred by clinicians or those involving prolonged antimicrobial therapy. In particular, the interventions included optimization of antimicrobial dosing, promotion of de-escalation, and determination of appropriate treatment duration. Post-prescription review and feedback was led by AST pharmacists, who extracted cases, reviewed electronic health records, and provided feedback to prescribing physicians via telephone or direct discussion. Recommendations for the management of SAB were provided in accordance with evidence-based care bundles ^(9)^.

We evaluated the impact of our ASP on inpatient antimicrobial use by comparing two periods: pre-intervention (April 2021-March 2022) and intervention (April 2022-September 2024). Monthly antimicrobial consumption was measured as days of therapy (DOTs) and antimicrobial use density (AUD) per 100 patient days for broad-spectrum agents. Specifically, DOTs and AUDs per 100 patient days were calculated for carbapenems (meropenem plus imipenem) and piperacillin/tazobactam. The system used for antibiotic data collection was based on the Japan Surveillance for Infection Prevention and Healthcare Epidemiology (J-SIPHE) platform ^(10)^.

For SAB, all consecutive hospitalized adult patients (≥18 years) with at least one positive blood culture for *S. aureus* were included. Demographic and clinical data were collected from medical records, including age, sex, onset setting (hospital-onset or not), microbiological findings, source of infection (e.g., catheter-related, endocarditis, osteomyelitis/arthritis, respiratory, skin and soft tissue, or unknown), and 14-day and 30-day mortality. Bundled care for SAB was defined based on evidence-based quality-of-care indicators (QCIs): (1) follow-up blood cultures within 7 days of the initial positive culture, (2) cardiac echocardiography to evaluate for infective endocarditis, (3) appropriate susceptibility-based antimicrobial therapy (e.g., cefazolin for methicillin-sensitive *Staphylococcus aureus*; vancomycin or daptomycin for methicillin-resistant *Staphylococcus aureus*), and (4) adequate treatment duration for at least 14 days. Patients with allergies to first-choice treatments, those receiving palliative care, patients not receiving any treatment, and those who died within 72 hours of blood culture collection were excluded. The methods were conducted in accordance with a previous study ^(11)^. Data were retrospectively reviewed from the electronic medical charts of AST members in MSGH.

Categorical data were analyzed using either the chi-squared test or Fisher’s exact test, and non-categorical data using the Student’s *t*-test or Mann-Whitney *U* test as appropriate. Interrupted time series (ITS) regression analysis was used to evaluate trends in monthly antimicrobial consumption before and after intervention ^(12)^. Statistical significance was defined at p < 0.05 with a 95% confidence interval (CI). Analyses were performed using R software (version 4.0.2).

## Results

During the study period, the total number of patient days at MSGH was 502,132, including 113,056 in the pre-intervention period (average: 9,421 per month) and 275,274 in the intervention period (average: 9,276 per month).

### Trends in the use of carbapenems and piperacillin/tazobactam

**Figure 1.**
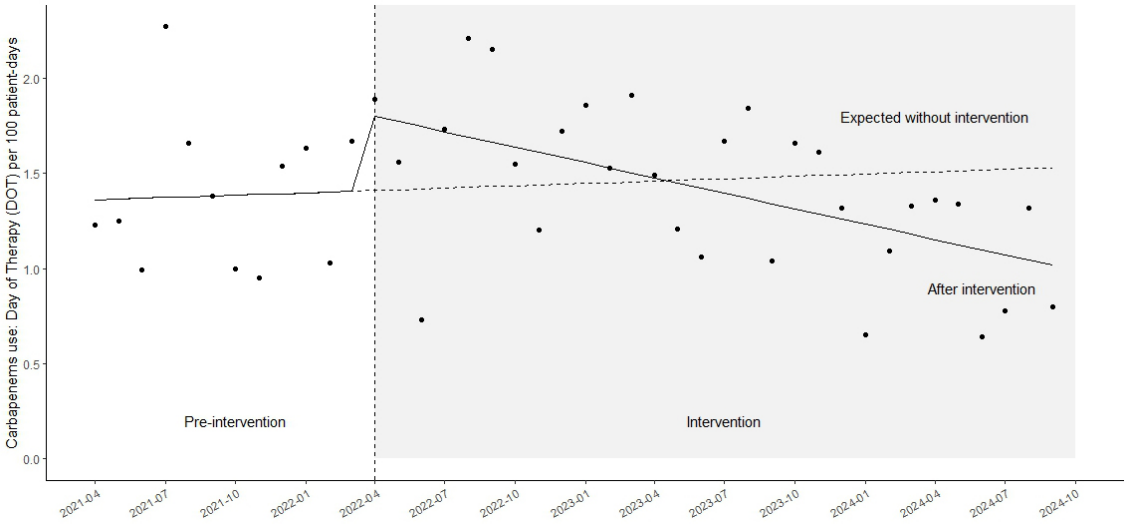
Time-series analysis of monthly antibiotic use. A time-series plot showing the monthly average duration of carbapenem use (DOTs/100 patient days). DOT: day of therapy.

The median monthly DOT per 100 patient days for carbapenems was 1.31 (interquartile range [IQR]: 1.01-1.65) during the pre-intervention period and 1.42 (IQR: 1.08-1.72) during the intervention period (p = 0.67). ITS analysis showed no immediate change (coefficient: 0.39; 95% CI: −0.15 to 0.93; p = 0.16), but a significant downward trend during the intervention period (coefficient: −0.03; 95% CI: −0.04 to −0.01; p < 0.01) (Figure 1). The median monthly AUD per 100 patient days for carbapenems was 0.88 (IQR: 0.78-1.24) during the pre-intervention period and 1.05 (IQR: 0.71-1.26) during the intervention period (p = 0.79). ITS analysis demonstrated a statistically significant immediate increase in carbapenem use following the intervention (coefficient: 0.73; 95% CI: 0.32-1.1; p < 0.01), as well as a significant downward trend during the intervention period (coefficient: −0.02; 95% CI: −0.02 to −0.01; p < 0.01). The median monthly AUD/DOT for carbapenems was 0.82 (IQR: 0.49-0.96) during the pre-intervention period and 0.67 (IQR: 0.54-0.98) during the intervention period (p = 0.93).

**Figure 2.**
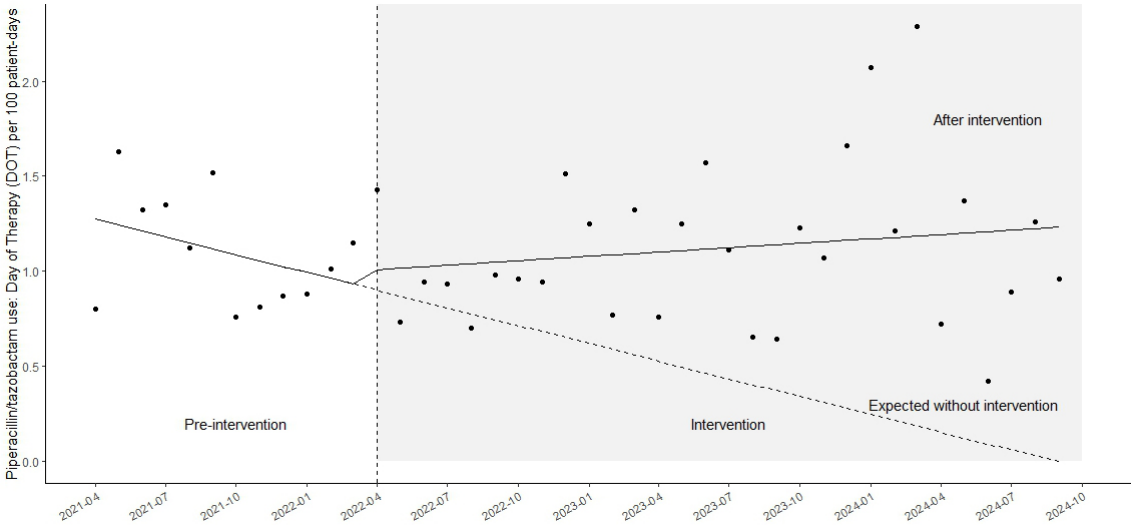
Time-series analysis of monthly antibiotic use. A time-series plot showing the monthly average duration of piperacillin/tazobactam use (DOTs/100 patient days). DOT: day of therapy.

The median monthly DOT per 100 patient days for piperacillin/tazobactam was 1.06 (IQR: 0.82-1.34) in the pre-intervention period and 1.02 (IQR: 0.77-1.33) during the intervention period (p = 0.88). No significant changes were observed in level (coefficient: 0.04; 95% CI: −0.03 to 0.11; p = 0.25) or trend (coefficient: 0.11; 95% CI: −0.45 to 0.67; p = 0.69) (Figure 2). The median monthly AUD per 100 patient days for piperacillin/tazobactam was 0.68 (IQR: 0.55-0.99) in the pre-intervention period and 0.76 (IQR: 0.57-0.99) during the intervention period (p = 0.63). No significant changes were observed in level (coefficient: 0.13; 95% CI: −0.26 to 0.51; p = 0.51) or trend (coefficient: 0.03; 95% CI: −0.01 to 0.08; p = 0.17). The median monthly AUD/DOT for piperacillin/tazobactam was 0.67 (IQR: 0.63-0.72) during the pre-intervention period and 0.70 (IQR: 0.67-0.75) during the intervention period (p = 0.10).

### Adherence to management bundles for SAB

A total of 56 patients with a first episode of SAB were included in the analysis, with 14 patients in the pre-intervention period and 42 patients in the intervention period. Baseline demographic characteristics and adherence to QCIs are summarized in Table 1. During the intervention period, there was a tendency for a higher proportion of female patients and a greater incidence of infective endocarditis. Overall, QCIs improved significantly during the intervention period, except for cardiac echocardiography. The intervention was independently associated with better adherence to follow-up blood cultures (14.3% - 69.1%), appropriate antimicrobial use (57.1% - 85.8%), and Intravenous treatment for at least 14 days (62.6% - 88.9%).

**Table 1.** Baseline Characteristics and Quality-Of-Care Indicator Adherence in Patients with *Staphylococcus aureus* Bacteremia during the Pre-Intervention and Intervention Periods.

Variables	Pre-intervention period		Intervention period		p Value
	n = 14	percent (%)	n = 42	percent (%)	
Age (years), median (IQR)	86.5 (81-92)	―	87 (78-91)	―	0.44
Sex female	9	64.2	15	35.7	0.06
					
Setting					
Hospital-onset	7	50.0	23	54.8	0.75
					
Microbiology					
MRSA	4	28.6	14	33.3	0.74
					
Source of bacteremia					0.14
Catheter-related bloodstream infections	3	21.4	11	26.2	
Endocarditis	0	0.0	5	11.9	
Osteomyelitis/arthritis	2	14.3	12	28.6	
Respiratory tract	0	0.0	1	2.4	
Skin and soft tissue infection	1	7.1	3	7.1	
Others	4	28.6	2	4.8	
Unknown source of bacteremia	4	28.6	8	19.1	
					
14-day mortality	3	21.4	6	14.3	0.53
30-day mortality	5	35.7	9	21.4	0.29
					
Adherence to quality-of-care indicators (QCIs)					
Follow-up blood culture on day 7	2/14	14.3	29/42	69.1	<0.01
TTE/TEE echocardiography	6/14	42.9	21/42	50.0	0.64
Appropriate antimicrobial therapy	8/14	57.1	36/44	85.8	0.02
Intravenous treatment for 14 days	7/11	63.6	32/36	88.9	0.05

IQR: interquartile range; MRSA: methicillin-resistant *Staphylococcus aureus*; QCI: quality-of-care indicator; TEE: transesophageal echocardiography; TTE: transthoracic echocardiography.

## Discussion

In this study, although there was no significant immediate change in carbapenem use following the intervention, a statistically significant downward trend was observed. Importantly, this reduction occurred without a compensatory increase in piperacillin/tazobactam use after the initiation of weekly part-time ID specialist collaboration with the antimicrobial stewardship team. Ideally, a reduction in the use of both carbapenems and piperacillin/tazobactam would have been preferable; however, this finding may reflect a limitation of part-time interventions. In contrast, the intervention may have contributed to more appropriate antimicrobial use. In this study, although both DOT per 100 patient days and AUD per 100 patient days showed a downward trend after the intervention, the significant immediate increase in AUD per 100 patient days may indicate a shift toward higher-dose antimicrobial therapy prompted by the intervention. According to data from the J-SIPHE for fiscal year 2023, DOT per 100 patient days and AUD per 100 patient days for carbapenems in hospitals included in this study (type 1) were reported as follows. The median DOT per 100 patient days was 1.99 (IQR: 1.09-2.92), and the median AUD per 100 patient days was 1.23 (IQR: 0.65-1.88) ^(13)^. In the present study, DOT per 100 patient days values were consistently lower than those reported by J-SIPHE. Further reductions are expected in the future.

This study demonstrated an improvement in adherence to the care bundle for SAB. In particular, the rate of follow-up blood cultures and appropriate antimicrobial therapy increased. Similar to previous studies in Japan, the rate of follow-up blood cultures was low prior to the intervention ^(11), (14)^. Management of SAB often depends on the involvement of AST and ID specialists. As a result, there may be variability in the quality of care across institutions, indicating potential areas for improvement. In Japan, in particular, in facilities without ID specialists, it may be necessary to foster a culture of routinely performing follow-up blood cultures as part of SAB management. In facilities without full-time ID specialists, the implementation of a hospital-wide protocol for SAB management may promote adherence to standard care ^(15)^. Therefore, such protocols should be introduced in medium-sized hospitals lacking ID specialists as well.

Although many studies have explored the optimization of antimicrobial use through stewardship programs, few have specifically examined the impact of part-time interventions, such as weekly participation. In Japan, one study reported that weekly attendance by ID physicians was associated with a reduction in broad-spectrum antibiotic use and positively influenced key parameters of ASPs ^(7)^. Another study found that part-time weekly involvement of ID physicians led to favorable outcomes in patient management, including a reduction in broad-spectrum antibiotics such as carbapenems and the successful implementation of ASP and quality improvement projects ^(8)^.

Considering the results of our study, collaboration between ID specialists and AST teams―even on a part-time basis―may support hospitals without full-time ID physicians, such as medium-sized community hospitals, and contribute to the successful implementation of antimicrobial stewardship. In our intervention, weekly on-site reviews were conducted during AST conferences, although alternative models such as asynchronous electronic consultations (eConsults) have also been reported. eConsultation offers a practical solution for hospitals without ID specialists and has been associated with lower 30-day mortality and high provider satisfaction ^(16), (17)^. Each model has its advantages and limitations. Importantly, regular case discussions during AST conferences not only support appropriate antimicrobial use but also serve as valuable opportunities for education and hands-on learning, thereby enhancing the overall capabilities of the AST team. Further research is needed to refine strategies for ID specialist involvement in antimicrobial stewardship, particularly in facilities without dedicated ID physicians.

In recent years, several pharmacist-led ASP initiatives have been reported in small- and medium-sized healthcare facilities in Japan ^(18), (19), (20)^. Implementing ASPs in these settings requires careful consideration of each facility’s unique context, leveraging the strengths of various healthcare professionals―including physicians, pharmacists, clinical laboratory technologists, and nurses―and promoting collaboration with other institutions to ensure efficient and effective antimicrobial stewardship.

This study has several limitations due to its retrospective, single-center design, which may limit the generalizability of the findings. Although an interrupted time-series analysis was conducted, the use of a pre-post design made it difficult to fully adjust for confounding factors such as time trends, patient characteristics, and the potential impact of the COVID-19 pandemic. Furthermore, the analysis focused exclusively on broad-spectrum antibiotics, thereby narrowing the scope of the results. In relation to SAB, this study focused on process indicators of the management bundle rather than assessing disease severity or other detailed clinical characteristics.

In conclusion, while carbapenem use showed no immediate change, a significant downward trend was observed with part-time ID specialist involvement in AST case reviews. No “see-saw effect” was seen for piperacillin/tazobactam. In addition, adherence to the care bundle for SAB, particularly follow-up blood cultures, improved after the intervention. These findings suggest that even part-time ID specialist participation can support antimicrobial stewardship in hospitals without dedicated ID services through active collaboration with AST teams.

## Article Information

### Acknowledgments

The authors thank the clinical staff at the Matsue Seikyo General Hospital for their excellent work.

### Author Contributions

Conceptualization: Yoshiro Hadano. Data curation: Yoshiro Hadano, Ryuichi Oku, Mika Yanagihara, and Sanae Sota. Formal analysis: Yoshiro Hadano. Methodology: Yoshiro Hadano and Ryuichi Oku. Project administration: Yoshiro Hadano, Ryuichi Oku, Mika Yanagihara, Kumiko Takahashi, and Yasuhiko Yokoyama. Supervision: Ryuichi Oku and Yasuhiko Yokoyama. Writing - original draft: Yoshiro Hadano. Writing - review and editing: all authors.

### Conflicts of Interest

None

### Approval by Institutional Review Board (IRB)

This study was approved by the MSGH Institutional Review Board (Number 202305) and conducted in accordance with the Declaration of Helsinki. The study only used data collected during clinical practice; therefore, the requirement for informed consent was waived owing to the retrospective design of the study.
